# Safety and Immunogenicity of a Candidate Bioconjugate Vaccine against Shigella flexneri 2a Administered to Healthy Adults: a Single-Blind, Randomized Phase I Study

**DOI:** 10.1128/CVI.00224-16

**Published:** 2016-12-05

**Authors:** Mark S. Riddle, Robert W. Kaminski, Claudio Di Paolo, Chad K. Porter, Ramiro L. Gutierrez, Kristen A. Clarkson, Hailey E. Weerts, Christopher Duplessis, Amy Castellano, Cristina Alaimo, Kristopher Paolino, Robert Gormley, Veronica Gambillara Fonck

**Affiliations:** a Naval Medical Research Center, Silver Spring, Maryland, USA; b Walter Reed Army Institute of Research, Silver Spring, Maryland, USA; c LimmaTech Biologics, Schlieren, Switzerland; University of Maryland School of Medicine

## Abstract

Several candidate vaccines against Shigella spp. are in development, but the lack of a clear correlate of protection from challenge with the induction of adequate immune responses among the youngest age groups in the developing world has hampered Shigella vaccine development over the past several decades. Bioconjugation technology, exploited here for an Shigella flexneri 2a candidate vaccine, offers a novel and potentially cost-effective way to develop and produce vaccines against a major pathogen of global health importance. Flexyn2a, a novel S. flexneri 2a bioconjugate vaccine made of the polysaccharide component of the S. flexneri 2a O-antigen, conjugated to the exotoxin protein A of Pseudomonas aeruginosa (EPA), was evaluated for safety and immunogenicity among healthy adults in a single-blind, phase I study with a staggered randomization approach. Thirty subjects (12 receiving 10 μg Flexyn2a, 12 receiving Flexyn2a with aluminum adjuvant, and 6 receiving placebo) were administered two injections 4 weeks apart and were followed for 168 days. Flexyn2a was well-tolerated, independently of the adjuvant and number of injections. The Flexyn2a vaccine elicited statistically significant S. flexneri 2a lipopolysaccharide (LPS)-specific humoral responses at all time points postimmunization in all groups that received the vaccine. Elicited serum antibodies were functional, as evidenced by bactericidal activity against S. flexneri 2a. The bioconjugate candidate vaccine Flexyn2a has a satisfactory safety profile and elicited a robust humoral response to S. flexneri 2a LPS with or without inclusion of an adjuvant. Moreover, the bioconjugate also induced functional antibodies, showing the technology's features in producing a promising candidate vaccine. (This study has been registered at ClinicalTrials.gov under registration no. NCT02388009.)

## INTRODUCTION

Shigellosis is a leading cause of diarrheal disease worldwide particularly in developing countries (1), and is also a continuing problem for civilian and military travelers visiting regions of endemicity (2, 3). Vaccine development remains a high priority given the disease burden (4, 5), increasing antibiotic resistance (6), and an increasing appreciation of the postinfectious sequelae associated with shigellosis (7, 8). Shigella flexneri accounts for 30 to 60% of shigellosis cases in developing regions, necessitating coverage of prevalent S. flexneri serotypes in a multivalent Shigella vaccine (9). Emerging data from studies where culture-independent diagnosis (e.g., via quantitative PCR [qPCR]) for Shigella was assessed indicate that traditional culture-based methods significantly underestimate the global burden of Shigella-associated illness (10). Estimates from the reanalysis of the Global Enteric Multicenter Study (GEMS) found that use of qPCR resulted in a 2- to 2.5-fold increase in the attributable fraction of Shigella-associated, moderate to severe diarrheal disease (10, 11).

Vaccines are considered an important component of an integrated strategy that also includes improved sanitation and hygiene, nutrition, and breastfeeding to reduce the Shigella-associated burden of disease in the developing world (12, 13) and, if available, would likely be used for travelers to the developing world (14, 15). Several vaccine approaches are under active investigation, including live-attenuated vaccines, inactivated whole-cell vaccines, subcellular vaccines, and purified subunit vaccines, such as the O-SP conjugate vaccines (8, 12). The lack of a clear correlate of protection, insufficient relevant disease animal models, and challenges with the induction of adequate mucosal immune responses among the youngest age groups in the developing world have hampered Shigella vaccine development over the past several decades (16–18). Even so, the importance of the serotype-specific LPS antigen is widely recognized and included as a component of all active vaccine approaches. An effective Shigella vaccine must not only consist of the appropriate antigens but also stimulate the protective immune response, which for shigellosis likely includes functional antibodies in the intestinal mucosal compartment. Also, given the invasive nature of the disease, an effective vaccine-induced systemic neutralizing response may be particularly important for the reduction of more severe invasive disease and dysentery. Relative to orally administered live-attenuated vaccine approaches, which have experienced challenges in safety and effectiveness when delivered to pediatric target populations in the developing world, conjugate vaccines have been demonstrated to be well-tolerated, to protect against a number of childhood diseases, and to have efficacy against shigellosis in field trials among adults and in older children (19, 20).

An initial phase I dose-escalation study evaluating the safety and immunogenicity of an S. dysenteriae bioconjugate demonstrated that the vaccine was safe and elicited strong humoral responses against the S. dysenteriae polysaccharide as well as functional antibodies against the protein carrier (21). In the current study, a Shigella flexneri 2a bioconjugate vaccine was evaluated to demonstrate reproducibility of this platform with a different O-antigen polysaccharide and the same protein carrier and to enable advancement to a human challenge model with a homologous S. flexneri 2a strain. Furthermore, the addition of aluminum adjuvant (alum) to the vaccine formulation was evaluated as part of our primary research objective focusing on safety and immunogenicity.

## MATERIALS AND METHODS

### Clinical trial design.

The study was conducted in one center and designed as a randomized single-blind study with the goal of enrolling 30 healthy adult volunteers. The primary study objective was to assess the safety and tolerability of two injections of 10 μg polysaccharide of the S. flexneri 2a bioconjugate vaccine Flexyn2a, administered alone or in combination with an alum adjuvant through study day 56. Secondary objectives included the following: (i) an evaluation of changes in hematological and biochemical safety parameters before (screening) and after administration of Flexyn2a vaccine, compared to the placebo group; (ii) comparison of the immune response induced by the Flexyn2a vaccine between baseline and after each injection; (iii) comparison of the immune response induced by the Flexyn2a vaccine alone or in combination with adjuvant at each postvaccination time point. Volunteers were randomized to three arms in which two dose formulations of Flexyn2a 10 μg (*n* = 12), Flexyn2a 10 μg in combination with Alum (*n* = 12), or placebo (*n* = 6) was given 28 days apart.

### Vaccine.

The candidate vaccine Flexyn2a has been developed with a recent technology called *in vivo* bioconjugation, which allows the biosynthesis of polysaccharide and carrier protein within Escherichia coli cells and the subsequent *in vivo* coupling of the proteins by using a specific oligosaccharyl transferase from the N-linked protein glycosylation system (22, 23). The Flexyn2a vaccine is delivered via an intramuscular injection as a suspension and is formulated in Tris-buffered saline (TBS; pH 7.4; 25 mM Tris, 137 mM NaCl, 2.7 mM KCl). The administered dose volume was 0.5 ml of Flexyn2a after on-site formulation with and without Alhydrogel adjuvant. Each dose contained 10 μg of S. flexneri 2a O-antigen polysaccharide and, if adjuvanted, 0.02% Al^3+^. The protein component amounts to approximately 50 μg EPA. This material has been characterized extensively using various standard analytical methods, including assays for content, purity, and structure (24).

### Study population and enrollment criteria.

Volunteers were healthy male and nonpregnant female adults between 18 and 50 years of age recruited from the Baltimore, MD–Washington, DC, greater metropolitan area and were enrolled after an informed consent process consisting of a detailed presentation of study material via a taped video, a comprehension test, and interview with an investigator. Volunteers were excluded from enrollment if they had a clinically significant acute or chronic disease, an immunosuppressive disorder or were on immunosuppressive medication, regularly used antidiarrheal, anticonstipation, or antacid therapy, had an abnormal stool pattern (<3 stools per week or >3 stools per day), had participated in other investigational product research, had a positive blood test for hepatitis B virus surface antigen, hepatitis C virus, or human immunodeficiency virus type 1, or had clinically significant abnormalities on basic laboratory screening. Based on epidemiological data and the identification of Shigella LPS isolated in the synovial fluid in reactive arthritis (25, 26), volunteers with a personal or family history of an inflammatory arthritis or positive blood test for HLA-B27 were excluded. Volunteers with a history of microbiologically confirmed Shigella infection, prior receipt of an experimental Shigella vaccine or live Shigella challenge, recent travel (within ≤2 years) to a country where Shigella or other enteric infections are endemic, or recent occupation (within ≤3 years) involving exposure to Shigella species were excluded. In addition, in an effort to maximize the inclusion of a Shigella-naive population, volunteers with a serum IgG endpoint titer of ≥2,500 to Shigella flexneri 2a LPS were excluded (threshold based on S. flexneri 2a LPS IgG titers previously obtained with volunteers from the Washington, DC, metropolitan area [data not shown]).

### Ethics.

This study was approved by the Institutional Review Board of the Naval Medical Research Center, Silver Spring, MD (DoD NMRC.2014.0018, WRAIR 2154) in compliance with all federal regulations governing the protection of human volunteers. The study is registered on ClinicalTrials.gov (registration number NCT02388009).

### Randomization, concealment, and immunization procedures.

Participants were randomized at a final ratio of 2:2:1 in a staggered approach. A first cohort of six subjects was randomized in a ratio of 1:1:1 to the three vaccination arms and assessed for safety after each injection. A second cohort of 24 subjects was allowed to be randomized in a ratio of 5:5:1 to the three vaccination arms after a safety assessment of the first cohort showed no safety concerns. Only volunteers were blinded to their group assignment. Depending on group assignment, a volunteer received two doses of the vaccine intramuscularly in the same arm 28 days apart.

### Safety monitoring.

Adverse event monitoring was conducted using in-person symptom surveillance, symptom memory aid, and targeted physical exams. Specifically, baseline clinical assessments, including symptom survey and local physical exam findings, were conducted at screening (up to 90 days prior to vaccination), 7 days prior to immunization, and on the day of immunization to record preimmunization findings. After each immunization, volunteers were observed for 30 min, after which a symptom survey and local physical exam were performed. A phone call to assess for safety after each dose was performed 24 h after each immunization. Daily for 7 days postvaccination, volunteers documented all adverse events (AE) in a memory aid. Volunteers returned to the clinic 7 days after each immunization for a focused exam and a memory aid review. Furthermore, blood for clinical safety assessment was drawn 7 days following each vaccine dose. The clinical observation period concluded 28 days after the second vaccine dose. Solicited adverse events included malaise, headache, or fever, as well as local vaccine site assessments of pain, tenderness, and redness. Information on any other symptoms, the use of any medications (prescription and/or over the counter), nonplanned medical consultations or doctor's visits, and hospitalizations were also collected. Severity of self-reported symptoms (adverse events) were recorded in accordance with Food and Drug Administration guidance on adverse event grading (27). In addition to grading severity of adverse events, the degree with which an AE could be attributed to administration of the test article was determined by the principal investigator (PI). All aspects of the trial were closely assessed by an independent medical research monitor who also monitored using the PI-established safety stopping criteria.

### Specimen collection, processing, and immunological assays.

Whole blood was collected prior to and during the vaccination period for immunological analysis and separated into peripheral blood mononuclear cells (PBMCs) for antibody lymphocyte secretion (ALS) on days 0, 7, 28, and 35 and serum on days 0, 28, and 56 for Shigella LPS-specific IgA and IgG titers and serum bactericidal activity (SBA). For ALS assays, freshly isolated PBMCs were suspended in complete RPMI medium (10% fetal calf serum, 1× penicillin-streptomycin [Pen-Strep], 1× glutamine) and plated in duplicate in a sterile 24-well tissue culture plate at 5 × 10^6^ cells per well. After culture for 4 days at 37°C with 5% CO_2_, supernatant was collected from both wells (1 × 10^7^ cells total) and frozen at −80°C until assayed in an enzyme-linked immunosorbent assay (ELISA).

Anti-S. flexneri 2a LPS-specific serum IgG and IgA antibody responses (28), as well as ALS samples, were assessed in ELISAs (29). Geometric mean titers (GMT) were determined for each group. If the determined serum titers were less than the starting dilution, a titer corresponding to half the starting dilution was assigned. Immune responders were defined *a priori* as having a ≥4-fold increase over the baseline titer.

SBA was evaluated as previously described for an opsonophagocytic killing assay (30), with minor modifications. Briefly, serum samples were serially diluted using 3-fold titrations in Hanks' balanced salt solution (HBSS) in a 20-μl/well volume. Samples were tested in duplicate in round-bottom 96-well plates (Corning Inc., Corning, NY). Ten microliters of bacterial suspension was added to each well, followed by 50 μl of 20% baby rabbit complement. Assay controls included wells with bacteria and complement only, as well as wells with bacteria and heat-inactivated complement only. Plates were incubated in a tissue culture incubator (37°C, 5% CO_2_) for 2 h. After incubation, plates were placed on ice for 10 min, and a 10-μl aliquot of the final reaction mixture was spot plated onto square 12- by 12-cm LB plates (Greiner Bio-One, Monroe, NC). Plates were tilted, and inoculum was allowed to run approximately 2 cm down each plate to distribute bacterial colonies. Plates were incubated overnight at 37°C, after which 25 μl of a 75% agar overlay containing 2,3,5,-triphenyltetrazolium chloride (TTC), a cellular metabolism-indicating dye that stains bacterial colonies red, was added to plates. The number of bacterial colonies in the agar plates was enumerated using NICE software, as previously described (31). SBA titers were defined as the reciprocal serum dilution that killed ≥50% of bacteria compared to controls. A titer of 3, corresponding to the lowest serum dilution tested, was assigned to samples not exhibiting detectable serum bactericidal activity. Immune responders were defined *a priori* as those with a ≥9-fold SBA titer increase over the baseline titer.

### Study outcomes and definitions.

The primary outcome was to assess the safety and tolerability of two consecutive injections of 10 μg polysaccharides of the S. flexneri 2a bioconjugate vaccine Flexyn2a alone or in combination with the adjuvant throughout the study. A secondary outcome was to evaluate postvaccination differences in the immune response, as defined by IgG and IgA antibody titers against S. flexneri 2a LPS, measured in an ELISA at day 0 (baseline), day 28 (1 month after the first dose), and day 56 (1 month after the second dose), between the 3 groups.

### Data analysis and statistical considerations.

Rates of all AEs were dichotomized as being either related (possible, probable, or definite) or not related (not related or unlikely) to the observed event during the follow-up period after vaccination and were analyzed to compare across intervention arms. For immunological analyses, qualitative (responder rates) and quantitative assessments were made in addition to evaluation of the kinetics of the immune response. Paired *t* tests were used to compare individual postvaccination responses to the baseline response within each treatment group. For immunological assessment, comparisons between groups were accomplished using a two-way analysis of variance (ANOVA) with a Bonferroni posttest (α = 0.05), by utilizing Prism for Mac (version 6.0). Unless otherwise specified, all statistical tests were interpreted in a two-tailed fashion using an α of 0.05.

As this was a phase I clinical trial, no formal sample size calculation was performed; however, each treatment group sample size (minimum *n* of 10) ensured that the probability of detecting at least one adverse event in the group was 80%, provided that the true adverse event rate exceeded 15%. Furthermore, using a binomial probability formula for no observed severe adverse events within the 24 volunteers receiving at least one dose of test article yielded a one-sided 97.5% exact confidence interval of 0 to 14%.

## RESULTS

### Participant flow.

**FIG 1:**
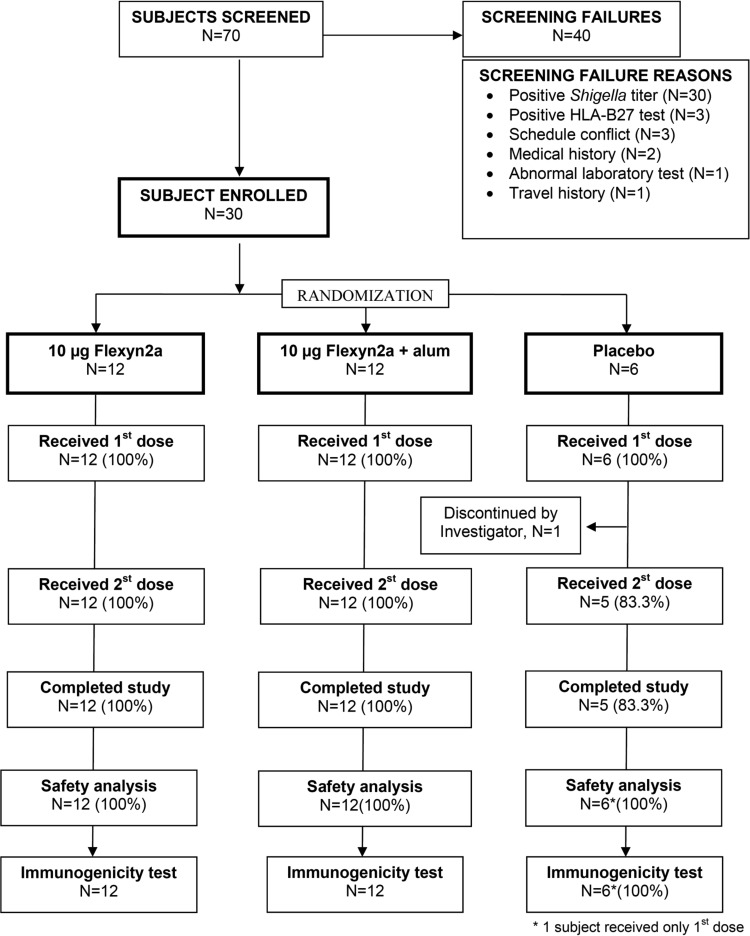
Study flow chart: subject screening, enrollment, and progress through the study.

A total of 70 volunteers signed a written informed consent and underwent prestudy screening, from which 30 subjects were enrolled and randomized (Fig. 1). The main reason for screening failure included an S. flexneri 2a LPS titer of ≥2,500 (*n* = 30 [43%]). The remaining 30 participants were randomized, and apart from one subject in the placebo group who was withdrawn due to follow-up noncompliance, they received two doses of study vaccine. The mean age of participants was 31 years (range, 19 to 50 years). There were no significant differences in gender (37% female) or race between vaccine groups (Table 1).

**TABLE 1:** Study population demographics

Characteristic	Value for vaccine group			
	Flexyn2a (*n* = 12)	Flexyn2a + alum (*n* = 12)	Placebo (*n* = 6)	Total (*n* = 30)
Age (yrs), mean (min–max)	30 (19–50)	31 (21–48)	32 (21–50)	31 (19–50)
Gender female, no. (%)	3 (25)	5 (42)	3 (50)	11 (37)
Ethnicity, no. (%)				
Hispanic or Latino	3 (25)	2 (17)		5 (17)
Non-Hispanic or non-Latino	9 (75)	10 (83)	6 (100)	25 (83)
Race, no. (%)				
Asian	1 (8)			1 (3)
Black	4 (33)	3 (25)	3 (50)	10 (33)
Multiracial	2 (17)	1 (8)		3 (10)
White	5 (42)	8 (67)	3 (50)	16 (54)

### Safety and clinical adverse events.

There were no serious adverse events or adverse events that met the stopping criteria. A slightly higher incidence of local adverse events (e.g., pain and tenderness) was observed in the group receiving the adjuvanted Flexyn2a candidate vaccine than in the other two groups (Tables 2 and 3). Subjects receiving adjuvanted Flexyn2a had a higher (nonsignificant) incidence of any adverse event (83%; 95% confidence interval [CI], 52 to 98%) than those who received Flexyn2a alone (75%; 95% CI, 43 to 95%), but all subjects in the placebo group experienced at least one adverse event (100%; 95% CI, 54 to 100%). Most (84%) of the AE were mild. Eleven percent of AE were moderate and mainly unrelated to the test article. A few severe AE unrelated to the vaccine were also observed (Table 2). The most frequent vaccine-related solicited AE were pain and tenderness, mainly mild in intensity, and the reactions resolved in a few days without intervention. There was a trend of increasing solicited local AE after the second injection, especially in the alum-adjuvanted group (Table 3). Two serious AE unrelated to the investigational product occurred during the clinical trial. One subject experienced an acute mental health event for which the subject was hospitalized. The subject made a complete recovery. Another subject experienced a grade 4 elevation of a liver function assay; this was found to be the result of alcohol and concomitant medication use. The subject was asymptomatic throughout, and liver function values returned to normal.

**TABLE 2:** Adverse event incidence, frequency, and severity for each study group

AE measure	Flexyn2a (*n* = 12)	Flexyn2a + alum (*n* = 12)	Placebo (*n* = 6)^*a*^
Overall AE incidence, no. (%)	9 (75)	10 (83)	6 (100)
Frequency of AEs	37	37	24
Severity, no (%)			
Mild	31 (84)	29 (78)	22 (92)
Not related	19 (51)	14 (38)	18 (75)
Related	12 (33)	15 (40)	4 (17)
Moderate	3 (8)	7 (19)	1 (4)
Not related	3 (8)	5 (14)	1 (4)
Related		2 (5)	
Severe	3 (8)	1 (3)	1 (4)
Not related	3 (8)	1 (3)	1 (4)
Related			

a One subject received only the first injection.

**TABLE 3:** Incidence of solicited local adverse events^*a*^

Type of AE	No. (%) in vaccination group with AE					
	Flexyn2a (*n* = 12)		Flexyn2a + alum (*n* = 12)		Placebo (*n* = 6)^*b*^	
	1st vacc	2nd vacc	1st vacc	2nd vacc	1st vacc	2nd vacc
Any AE	3 (25)	5 (42)	4 (33)	7 (58)	2 (33)	
Pain	2 (17)	2 (17)	1 (8)	3 (25)		
Tenderness	2 (17)	4 (33)	3 (25)	7 (58)	2 (33)	
Redness						
Swelling		1 (8)				

a AE data were collected for subjects after the first and second vaccinations (vacc).

b One subject received only the first injection.

### Immunological responses. (i) Antigen-specific serum IgG and IgA.

**FIG 2:**
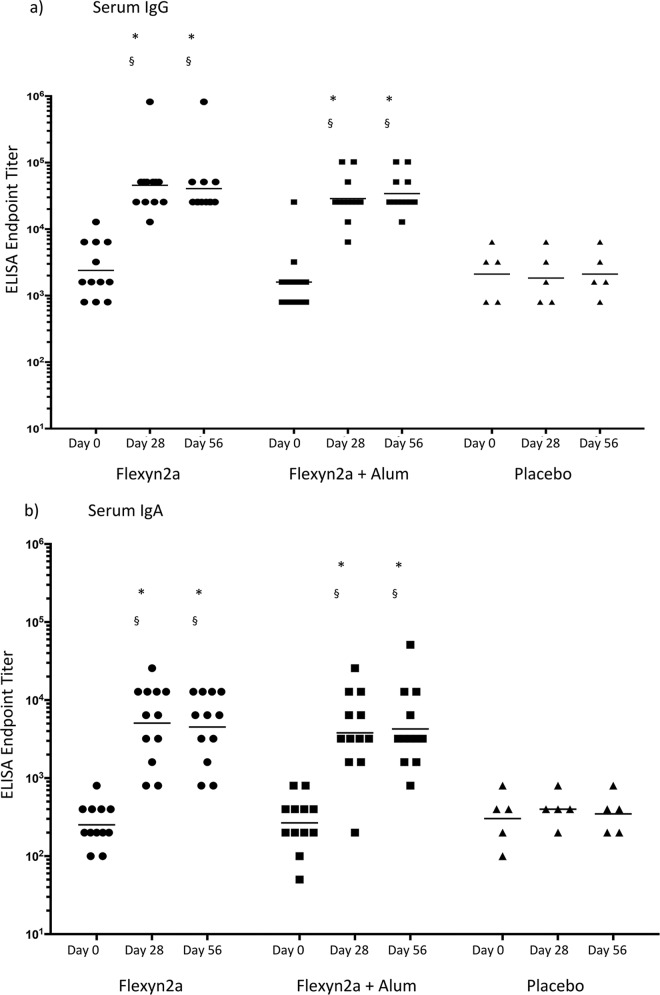
S. flexneri 2a LPS-specific serum IgG (a) and IgA (b) responses. ELISA endpoint titers over time are shown for day 0 (baseline), day 28 (post-first vaccination), and day 56 (post-second vaccination), with geometric means. §, statistically significantly different from baseline samples; *, statistically significantly different from placebo at the same time point.

Intramuscular immunization with 10 μg of Flexyn2a with or without alum induced robust anti-S. flexneri 2a LPS serum immune responses (Table 4). For a few subjects, the baseline titer (day 0) was higher than the expected threshold of 2,500 (due to different sample dilution steps in the ELISA method used at screening versus at day 0 [data not shown]). However, there were no appreciable differences in the fold increases over baseline titers among volunteers with different titers prevaccination. In addition, no statistical difference in baseline titers was observed between groups. Samples collected from placebo controls on day 28 and day 56 did not demonstrate any increase over the course of the trial. In contrast, immunization with Flexyn2a (with or without adjuvant) elicited a 16-fold or greater increase in anti-S. flexneri 2a LPS serum IgG compared to baseline samples. Both vaccine groups also had significantly higher peak GMT (*P* < 0.0001) than the placebo group on day 28 and day 56. Anti-S. flexneri 2a LPS serum IgA responses showed similar kinetics as the serum IgG responses, although the responses were lower in magnitude. Both vaccine groups showed a 14-fold or greater increase in serum IgA over baseline and had significantly higher peak GMT (*P* ≤ 0.0015) compared to the placebo group. The vaccine elicited a high number of subjects with S. flexneri 2a LPS-specific serum IgG and IgA seroconversion, with 92 to 100% of the volunteers showing a ≥4-fold increase in serum titers by day 56. Additionally, there were no significant differences between the LPS-specific serum antibody responses after immunization with adjuvanted or nonadjuvanted Flexyn2a, nor was there an observed increase in the magnitude of the serum titers between the first and second dose of the vaccines for either the IgG or IgA response (Table 4 and Fig. 2).

**TABLE 4:** Geometric mean titers of S. flexneri 2a (Sfl2a) LPS specific IgG and IgA serum, ALS and SBA titers over time and their within-group comparisons (*P* values) at each post-vaccination visit compared to baseline

Immune response and sample day	Vaccination group							
	Flexyn2a (*n* = 12)			Flexyn2a + alum (*n* = 12)			Placebo (*n* = 5)^*a*^	
	GMT	Responders^*c*^	*P* value^*d*^	GMT	Responders	*P* value	GMT	Responders
Anti-Sfl2a LPS Serum IgG								
Day 0	2,397		NS	1,600		NS^*e*^	2,111	
Day 28	45,614	11/12	<0.0001	28,735	12/12	<0.0001	1,838	0/5
Day 56	40,637	11/12	<0.0001	34,172	12/12	<0.0001	2,111	0/5
Anti-Sfl2a LPS Serum IgA								
Day 0	252		NS	267		NS	303	
Day 28	5,080	11/12	0.0002	3,805	11/12	0.0015	400	0/5
Day 56	4,525	11/12	0.0002	4,271	12/12	0.0002	348	0/5
Anti-Sfl2a LPS ALS IgG								
Day 0	1		NS	1		NS	1	
Day 7	48	12/12	<0.0001	30	10/12	0.0001	1	0/4^*b*^
Day 28	1	1/12	NS	1	0/12	NS	1	0/5
Day 35	5	7/12	NS	8	10/12	NS	1	0/5
Anti-Sfl2a LPS ALS IgA								
Day 0	1		NS	1		NS	1	
Day 7	17	8/12	0.001	19	12/12	0.0005	1	0/4
Day 28	1	0/12	NS	1	0/12	NS	1	0/5
Day 35	2	2/12	NS	3	5/12	NS	1	1/5
Serum bactericidal assay								
Day 0	29		NS	50		NS	201	
Day 28	391	8/12	NS	1188	9/12	NS	145	0/5
Day 56	817	10/12	0.0298	1430	10/12	0.0027	51	0/5

a Six subjects enrolled in the study. One subject dropped out post-first vaccination and was not included in the immunological analysis.

b One subject was unable to comply at the day 7 visit.

c “Responder” was defined as those with a 4-fold increase over baseline for serum and ALS responses and a 9-fold increase over baseline for bactericidal responses.

d *P* values were determined using a two-way ANOVA with a Bonferroni multiple comparison posttest (α = 0.05) of log-transformed titers in comparison to placebos at the same time point.

e NS, not significant.

### (ii) LPS-specific IgG and IgA in ALS.

PBMCs, isolated at various time points throughout the course of the study, were evaluated for S. flexneri 2a LPS-specific antibody secretion (Table 4). Peak S. flexneri 2a LPS-specific IgG ALS titers were observed 7 days after the first vaccination with Flexyn2a and Flexyn2a plus alum in 100% and 83% of volunteers, respectively. Both groups had significantly higher GMT (*P* ≤ 0.001) than the placebo group on day 7. Similarly, day 7 was the peak for S. flexneri 2a anti-LPS-specific IgA in ALS, with 67% and 100% of responders in the groups immunized with Flexyn2a and Flexyn2a plus Alum, respectively. Again, both vaccine groups had statistically higher GMT (*P* ≤ 0.001) than the placebo-immunized group. There were no significant differences between the groups immunized with or without adjuvant, although the study was not powered to detect such differences. The remainder of the LPS-specific ALS responses were low to undetectable on days 28 and 35, with the exception of IgG responses on day 35 in the group immunized with Flexyn2a plus alum (Table 4).

### (iii) Functional antibody responses.

**FIG 3:**
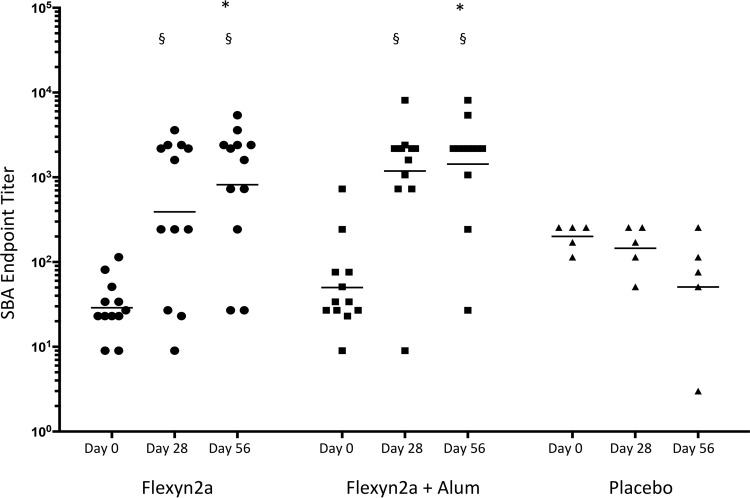
Anti-S. flexneri 2a strain 2457T serum bactericidal endpoint titers (with geometric means) of vaccinated subjects over time: day 0 (baseline), day 28 (post-first vaccination), and day 56 (post-second vaccination). §, statistically significant difference from baseline samples; *, statistically significant difference from placebo at the same time point.

Serum samples collected on days 0, 28, and 56 were evaluated in an S. flexneri 2a bactericidal assay to determine functional antibody responses induced after vaccination (Table 4 and Fig. 3). Volunteers with a 9-fold increase in the SBA titer over baseline were considered responders. After the first immunization, both groups immunized with the Flexyn2a vaccines had a higher number of responders than did placebo controls and significantly higher SBA titers than the respective baseline titers (Flexyn2a, 8/12; Flexyn2a plus alum, 9/12; placebo, 0/5). After the second immunization, the SBA titers on day 56 increased in both groups that received the Flexyn2a vaccines (Flexyn2a, 10/12; Flexyn2a plus alum, 10/12) and these were significantly higher than titers in the placebo control group (*P* ≤ 0.0298). There was no significant difference in the number of responders or magnitude of the SBA titers between groups immunized with adjuvanted or nonadjuvanted vaccine at either time point assayed postvaccination (Fig. 3).

## DISCUSSION

This S. flexneri 2a vaccine is the second Shigella bioconjugate vaccine to be tested in humans (21) and was evaluated as a potential component of a multivalent Shigella vaccine under development via bioconjugation technology. In addition to S. flexneri 2a, the multivalent candidate vaccine will contain the O-antigens of Shigella sonnei and S. flexneri 3a and 6, in order to broadly protect against Shigella disease in both pediatric and traveler target populations (9).

The primary objective of the study was to assess the safety profile of the S. flexneri 2a bioconjugate vaccine in humans. The safety analysis of Flexyn2a in this trial confirmed the initial safety observations obtained with the SD133 vaccine (against Shigella dysenteriae) (32) and did not generate any safety concerns. We did note a slightly higher incidence of local adverse events (e.g., pain and tenderness) in the group receiving the adjuvanted Flexyn2a candidate vaccine and slightly higher local adverse event reporting after the second dose in each of the groups than observed in the placebo group. The incidence and intensity of the observed local and general adverse events were mostly mild and self-limiting in nature and comparable to the safety profiles of licensed conjugate vaccines (i.e., Menveo, Prevenar13, Nimenrix, and Synflorix).

The secondary objective of this study was to assess immunological responses elicited after a primary and booster immunization as well as the effect of adjuvant on the number of vaccine responders, the magnitude of the serum titers, and generation of functional antibody responses. Flexyn2a was able to elicit a robust anti-S. flexneri 2a LPS-specific IgG and IgA response that peaked after the first vaccination. While not statistically powered to detect such differences, between-group analysis of active vaccination arms revealed no significant differences between the serum endpoint titers achieved with Flexyn2a alone and those achieved with Flexyn2a in combination with an adjuvant, indicating no benefit of the aluminum adjuvant. The lack of significant differences in the magnitudes of the IgG and IgA responses after the first and second vaccinations suggests that a single dose may provide adequate immunity. However, there was a significant difference in SBA titers, suggesting that although a booster immunization may not affect the magnitude of the response, a second dose may play a substantial role in development of functional antibodies. However, a range of SBA titers was induced in the vaccinated groups, highlighting that even though the vaccine elicited a robust antigen-specific IgG response, the functionality of antibodies can differ quite substantially between subjects and requires further study and validation in challenge studies. These findings should be further evaluated in larger studies and different populations to fully elucidate the contribution(s) of multiple doses to the affinity, avidity, and functionality of the antibody response induced after vaccination.

Unfortunately, there are no validated immune correlates or surrogates of protection to guide the rational development of Shigella vaccines. The current paradigm for protection against Shigella infection considers the potential need for both systemic and mucosal responses directed to the serotype-specific Shigella O antigens and conserved invasion plasmid antigens (IpaB, IpaC, and IpaD). In addition, antigen-specific B memory (B_M_) responses and antigen-specific antibody-secreting plasma cells in the peripheral circulation may play a key role for a protective systemic and mucosal response (16). Circulating B cells were isolated 7 days after each immunization to identify newly activated B cells producing 2a antibodies. Analysis of ALS samples demonstrated that the majority of responses were detected 7 days after the primary immunization, compared with 7 days after the second vaccination. The lack of a detectable response after the second vaccination may be a result of suboptimal timing of sample collection, but it may also be the result of activated B cells homing to mucosal sites. A protective role of the mucosal immune response against Shigella infection has been previously proposed (16). However, confirmatory data from natural infection or challenge studies are currently missing. As the homing kinetics of B cells after parenteral immunization with a conjugate vaccine are largely unknown, additional analyses are under way to understand the homing potential of LPS-specific B cells with relation to mucosal cell adhesion markers.

While traditional immunoassays have been established to discern humoral responses, as reported here, functional assays that correlate with clinical severity and immunological status indicative of vaccine effectiveness are needed in the field. In collaboration with the University of Alabama, Birmingham, the Subunit Enteric Vaccines and Immunology group at Walter Reed Army Institute of Research has developed an SBA for the potential evaluation of vaccine efficacy in human challenge and clinical field trials. Similarly, preliminary data from Pasetti et al. at the Center for Vaccine Development have recently reported correlations with an SBA under development and measures of disease severity and clinical outcome (33). In this study, the candidate vaccine Flexyn2a was able to induce functional antibodies with and without the addition of adjuvant, compared to the preimmune serum. Placebo-immunized individuals did not generate any bactericidal activity changes. However, the heterogeneity in the Flexnyn2a groups highlights the need for further investigation in expanded trials. Further work is necessary to validate the SBA as a reliable correlate of protection and to correlate IgG subclasses with SBA titers.

The need for a Shigella vaccine in both the developing world and for traveler populations continues to grow. In a reanalysis of samples from GEMS, the application of quantitative PCR resulted in a near-2-fold increase in Shigella detection rate in cases of moderate to severe diarrhea among children under 5 years (10). A more extensive reanalysis of GEMS samples by Platts-Mills et al. found that the increase in attributable risk was 2- to 2.5-fold higher when quantitative culture-independent (PCR) testing was used, compared to traditional methods (11). These data should result in a reconsideration of the disease burden and provide a reemphasis of the value that a safe and effective Shigella vaccine could have on global public health through reductions in mortality and morbidity, particularly in children under 5 years, but also in older children and adults (9, 34).

In travelers, there are currently no alternatives other than to treat individuals who develop moderate to severe disease. However, growing evidence supporting the link between shigellosis and chronic health consequences (e.g., reactive arthritis and irritable bowel syndrome) (35, 36), the association between travelers' diarrhea (TD) (treated and untreated) and colonization with extended-spectrum beta-lactamase-producing Enterobacteriaceae (37), as well as concern for multidrug-resistant strains of Shigella, further complicate treatment (6, 38, 39), making primary prevention a desired goal. More studies are needed to better understand the true prevalence of Shigella among travelers, with application of culture-independent diagnostic techniques. If the increase in Shigella burden through application of these methods approximates those found in the developing world's pediatric populations, a strong case could be made for the consideration of a Shigella vaccine by travelers for not only the protection from acute disease but also the costly and burdensome chronic consequences.

The field of Shigella vaccine development has developed significantly over the past several years (12, 18, 40, 41). One of the most promising approaches was the development of chemical conjugates that were safe, immunogenic, and able to induce protection in adults and young Israeli children (over age 4 years), as well as in Israeli soldiers in field trials (20). However, one of the obstacles of chemically synthesized conjugate vaccines is that their manufacture is complex and expensive, as it requires biological as well as chemical steps. Moreover, due to the nonspecific nature of the chemical linkage, the resulting vaccine was heterogeneous, variable from batch to batch, and produced via a relatively low yield. LimmaTech (previously GlycoVaxyn) has developed a novel technology that enables the *in vivo* biosynthesis of bioconjugate vaccines by directly linking the polysaccharides to the carrier protein using engineered bacterial cells (24). This process allows *in vivo* conjugation of bacterial polysaccharides to specific consensus sites for N-glycosylation of any carrier protein and removes the requirement for chemical detoxification of LPS and further conjugation processes, in a potentially cost-effective manner.

### Conclusion.

The results from this trial have provided necessary data to advance the development of the bioconjugate candidate vaccine Flexyn2a. The acceptable safety profile combined with the robust immune response necessitates moving forward into a phase IIb challenge study (proof of concept) with S. flexneri 2a strain 2457T to evaluate vaccine efficacy. If efficacious, a multivalent Shigella vaccine capable of providing immunological protection against S. flexneri 2a, 3a, and 6, S. sonnei, and potentially S. dysenteriae will be produced utilizing current manufacturing processes. Further studies evaluating vaccine dosing and regimens will also be required as these products move through the vaccine development pipeline into target populations of the developing world.
